# Broad-Specificity mRNA–rRNA Complementarity in Efficient Protein Translation

**DOI:** 10.1371/journal.pgen.1002598

**Published:** 2012-03-22

**Authors:** Pamela A. Barendt, Najaf A. Shah, Gregory A. Barendt, Casim A. Sarkar

**Affiliations:** 1 Department of Bioengineering, University of Pennsylvania, Philadelphia, Pennsylvania, United States of America; 2 Genomics and Computational Biology Graduate Group, University of Pennsylvania, Philadelphia, Pennsylvania, United States of America; 3 Perelman School of Medicine Information Services, University of Pennsylvania, Philadelphia, Pennsylvania, United States of America; 4 Department of Chemical and Biomolecular Engineering, University of Pennsylvania, Philadelphia, Pennsylvania, United States of America; Uppsala University, Sweden

## Abstract

Studies of synthetic, well-defined biomolecular systems can elucidate inherent capabilities that may be difficult to uncover in a native biological context. Here, we used a minimal, reconstituted translation system from *Escherichia coli* to identify efficient ribosome binding sites (RBSs) in an unbiased, high-throughput manner. We applied ribosome display, a powerful *in vitro* selection method, to enrich only those mRNA sequences which could direct rapid protein translation. In addition to canonical Shine-Dalgarno (SD) motifs, we unexpectedly recovered highly efficient cytosine-rich (C-rich) sequences that exhibit unmistakable complementarity to the 16S rRNA of the small subunit of the ribosome, indicating that broad-specificity base-pairing may be an inherent, general mechanism for efficient translation. Furthermore, given the conservation of ribosomal structure and function across species, the broader relevance of C-rich RBS sequences identified through our *in vitro* evolution approach is supported by multiple, diverse examples in nature, including C-rich RBSs in several bacteriophage and plants, a poly-C consensus before the start codon in a lower eukaryote, and Kozak-like sequences in vertebrates.

## Author Summary

In order to maintain an appropriate balance of proteins in the cell, the protein factories (ribosomes) translate different messages (mRNAs) into protein at different rates. Many human diseases, including cancer and certain hereditary diseases, are caused by making too much or too little protein. Additionally, infections caused by bacteria and viruses are enabled by the ability of these organisms to produce protein very quickly while situated in their host. For these reasons, it is important to understand the ways in which ribosomes may recognize mRNAs and initiate translation into protein. We developed an experimental system that allowed us to uncover the inherent mRNA–binding ability of the ribosomes in a common bacterium, *Escherichia coli*. We found evidence that, when removed from the native cellular environment, these ribosomes are able to make protein very efficiently using previously unidentified ribosome binding sites on the mRNA that closely resemble known ribosome binding sites in diverse organisms, including plants and humans. Our results suggest a general, ubiquitous mechanism of mRNA–ribosome association during translation initiation.

## Introduction

The ribosome is widely recognized as a broad-specificity ribozyme that is able to translate mRNA at different rates to maintain appropriate relative protein levels and thereby fulfill the dynamic needs of the cell [1]–[3]. Problems with increased or decreased translation of certain messages are known to lead to cancer and various other hereditary diseases in humans [4]. One of the major determinants of translational efficiency is the 5′ untranslated region (5′ UTR), which may contain a canonical RBS such as the Shine-Dalgarno (SD) sequence [5] in prokaryotes or the Kozak sequence [6] in vertebrates. Recently, it has been noted that, while the SD consensus sequence (5′-GGAGGU-3′) is generally an important cue for ribosome binding in prokaryotes, there are actually more non-SD-led genes than SD-led genes in some microbial genomes [7]. Additionally, the Kozak sequence is a relatively weak consensus, as only a very small fraction of vertebrate genes (∼0.2%) have the exact GCCGCC(A/G)CCAUGG sequence [8]. These observations do not immediately suggest a universal answer to the following fundamental question: what 5′ UTR sequences *inherently* enable a ribosome to bind mRNA, initiate translation, and proceed to elongation as quickly as possible?

Although efficient RBSs have been previously identified by library approaches both *in vivo* [9], [10] and in cell extracts *in vitro* [11], [12], the mechanisms of efficient translation are confounded by the multitude of uncharacterized biomolecular interactions in these environments. Furthermore, both the library size and the sequencing throughput in earlier studies have been limited, hindering identification of statistically significant motifs. To more directly answer the question posed above, we performed selections on a large RBS library (∼3.7×10^13^ mRNA molecules; ∼6.9×10^10^ unique sequences) in a minimal, well-defined, *E. coli*-based translation system [13]–[15] using ribosome display [16]. By using a minimal translation system, we removed unnecessary confounding variables and took a “bottom-up” approach to address the question of what sequences inherently promote the fastest translation.

One of the major goals of synthetic biology is to reveal new fundamental biological insights through the use of well-defined systems. The present study complements previous advances in the field that utilized or focused on differential RBS function, including work on riboregulators [17]–[19] and the RBS Calculator [20], as well as early work on synthetic gene networks that used RBSs of various strengths to adjust the gene expression dynamics of synthetic constructs [21]. Here, we were able to attribute the selected RBSs directly to the contents of the translation system because of its fully defined nature; additionally, we were able to consider general aspects of RBSs, which are not necessarily *E. coli*-specific, as the basic translational machinery is highly conserved across species.

High-throughput sequencing of the library after stringent selection for translational efficiency surprisingly revealed mostly non-SD motifs. These library members, some of which were nearly as efficient as the SD-containing 5′ UTR sequence derived from enterobacteriophage T7 gene 10, were generally highly C-rich. While it is well appreciated that SD sequences help to form the preinitiation complex by binding to the anti-SD sequence in the unpaired 3′ end of the 16S rRNA in the 30S ribosomal subunit, we further hypothesized that our efficient non-SD RBSs also achieve fast translation by optimizing binding to the 16S rRNA. (“Fast translation” in our study should be considered rapid in the context of the minimal system; the potential speed of translation may be much higher *in vivo*.)

Based on statistical analyses and competition studies, we conclude that base-pairing between the short, C-rich motifs of the non-SD RBSs and the G-rich rRNA of the small ribosomal subunit allows for fast translation, most likely through fast repositioning of the mRNA on the small ribosomal subunit to form a productive preinitiation complex that is then able to join the large ribosomal subunit and proceed quickly to elongation. We have demonstrated that pure poly-cytosine (poly-C) is a poor RBS, and we have used rational mutagenesis to show that the specific positioning of non-C nucleotides in a C-rich context is a critical determinant of translational efficiency. We also show that the activity of C-rich RBSs, but not SD RBSs, can be strongly decreased *in vitro* by the addition of random oligonucleotide competitor sequences, which can explain their differential activities *in vivo*. Furthermore, we report similarities between the most common motifs in our selected RBSs and those in human RBSs, suggesting that structurally and functionally conserved ribosomes from diverse organisms are inherently capable of utilizing C-rich sequences directly upstream of AUG start sites. The broader relevance of C-rich RBSs is further supported by several other examples in nature, including C-rich RBSs in non-*E. coli* bacteriophage, C-rich RBSs that base-pair to a G-rich rRNA element in plants [22], [23], and a poly-C consensus before the start codon in a lower eukaryote [24]. The overall goal of this study was to determine inherent requirements for fast translation, and our experimental and computational results together provide evidence of a general, broad-specificity mechanism for efficient protein synthesis.

## Results

### Enrichment of RBSs that promote fast translation in a minimal system

**Figure 1:**
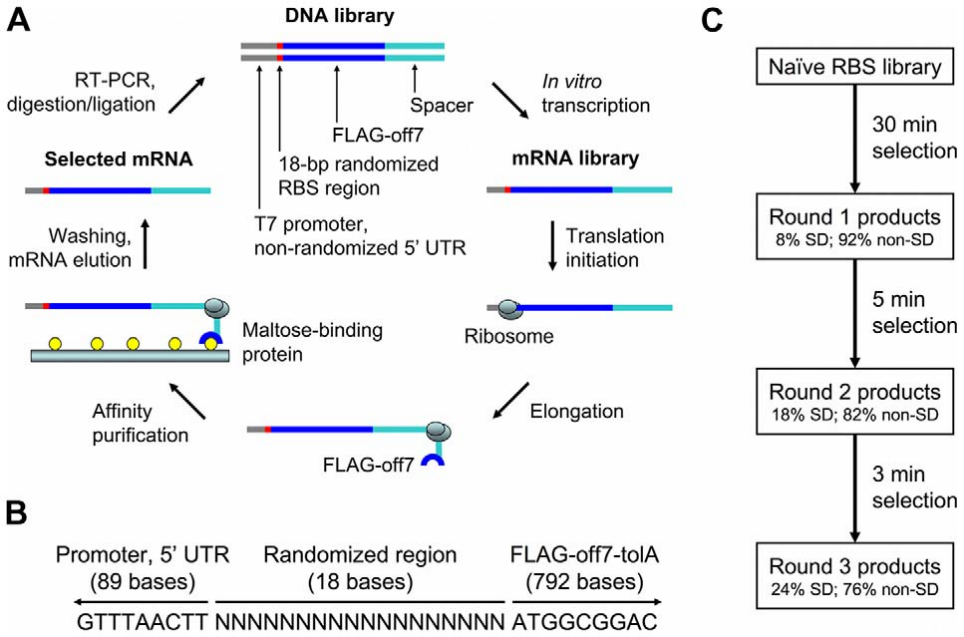
Ribosome display, library context, and selection scheme. (A) Our adaptation of ribosome display for selection of efficiently translated sequences is shown. The naïve DNA library contained an 18-bp randomized RBS region prior to the start codon. Selection was performed by limiting the time of *in vitro* translation. Multiple rounds of increasing stringency were performed. (B) The context of the randomized RBS region is shown. Upstream is the T7 promoter and 5′ UTR of the ribosome display vector, pRDV, which is partially derived from phage. This region contains 89 bases in the DNA construct and 63 bases in the mRNA transcript (5′ UTR only). Downstream is a fusion protein consisting of a FLAG tag, off7 (a designed ankyrin repeat protein which binds maltose-binding protein), and tolA (an unstructured spacer derived from *E. coli* tolA which allows off7 to exit the ribosomal tunnel and fold properly). There is no stop codon. (C) The basic selection scheme is shown. The naïve RBS library was subjected to three selection rounds of increasing stringency: 30 min, 5 min, and 3 min translation. SD sequences were moderately enriched between rounds, but many non-SD sequences remained in the pool after the highly stringent 3 min translation.

To investigate what upstream sequences promote fast translation, we chose a minimal, reconstituted, *E. coli*-based *in vitro* translation system: PURExpress (New England Biolabs) developed from PURE technology [13], [25], [26]. Ribosome display has previously been used to evolve peptides and proteins with desirable properties, including enhanced affinity and stability [16], [27]–[29]. Briefly, the standard method involves multiple cycles of generating a DNA library, *in vitro* transcription, *in vitro* translation, selection through binding, and recovery. The mRNA contains, at minimum, an RBS followed by a region encoding the gene of interest and an unstructured protein spacer with no stop codon, so that the ribosome stalls at the end of the mRNA, forming an mRNA-ribosome-polypeptide complex (hereafter called a ribosomal complex). In our adaptation (Figure 1A), we used a randomized 5′ UTR (Figure 1B) and progressively shortened the translation time in each round to impart an increasing selection pressure.

The 5′ UTR from the ribosome display vector pRDV [30] was considered the wild-type (WT) sequence. It includes a 5′ stem-loop to prevent degradation and a translational enhancer and SD RBS derived from enterobacteriophage T7. In the library version, the 18 nucleotides just prior to the start codon (5′-TAAGAAGGAGATATATCC-3′ in WT; SD sequence underlined) were fully randomized, creating a theoretical diversity of 4^18^ = 6.9×10^10^ different sequences, which can be nearly exhaustively sampled in our *in vitro* system. The SD sequence, when present, generally has a context-dependent optimal position within this region [31]. Additionally, previous studies investigating the position of mRNA on the 30S ribosomal subunit have suggested that approximately 15 bases prior to the start codon are protected by the ribosome during initiation [32], making this a region of particular interest. The invariant coding region was chosen to be a fusion protein containing (from N- to C-terminus) an initiating Met, Ala, FLAG-tag, Gly-Ser (BamHI site), off7 [30], Lys-Leu (HindIII site), and a modified version of the pRDV tolA spacer that contains out-of-frame stop codons. Off7 is a designed ankyrin repeat protein (DARPin) that was evolved to bind maltose-binding protein of *E. coli* with nanomolar affinity (∼4.4 nM) [30]. We chose this model protein because it translates and folds well *in vitro*. Additionally, its high affinity for maltose-binding protein enables easy affinity purification of only those ribosomal complexes with fully translated protein.

We performed three rounds of selection (30 min, 5 min, and 3 min translation at 37°C; the “30-5-3 selection”) and, despite increasingly stringent translation times, the number of recovered mRNA molecules climbed from ∼4.4×10^9^ in the first round to ∼1.5×10^10^ in the second round to ∼2.2×10^10^ in the third round. Quantitative reverse transcription-PCR (qRT-PCR) data and accompanying experimental details are presented in Figure S1. mRNA recovery from the third round was comparable to that produced from the WT pRDV RBS, which is highly efficient both *in vitro* and *in vivo*. This third round pool was subjected to in-depth analysis.

### RBSs that promote fast translation are predominantly non-SD and C-rich

We sequenced the enriched pools from each round in the 30-5-3 selection using the Roche 454 platform. Approximately 7,000 raw sequences were obtained from each round: 7,268 from round 1; 6,825 from round 2; and 7,525 from round 3. Sequences were excluded from analysis if they did not have 18 bases in the randomized region, if they included an in-frame AUG within the randomized region that could serve as an alternate start site, or if there were errors in the 10 bases on either side of the randomized region. Approximately 5,000 sequences were analyzed from each round: 5,202 (4,933 unique) from round 1; 4,880 (4,586 unique) from round 2; and 4,863 (4,551 unique) from round 3. SD sequences were broadly defined as any sequence containing one of the following four-base motifs which could base-pair to the 3′ tail of the 16S: AAGG, AGGA, GGAG, GAGG, and AGGU. The overall incidence of SD motifs in each round is shown in Figure 1C. The positional and overall frequencies of each individual SD motif at the end of each round are presented in Figure S2. In our data, the first G of GGAG is enriched most prevalently around position −12, while the same nucleotide is favored around position −10 in *E. coli* [31]. Certainly, mRNA context may affect the optimal position of SD motifs, as may different *in vitro* or *in vivo* conditions. Position-dependent enrichment of SD motifs validated our selection method.

**Figure 2:**
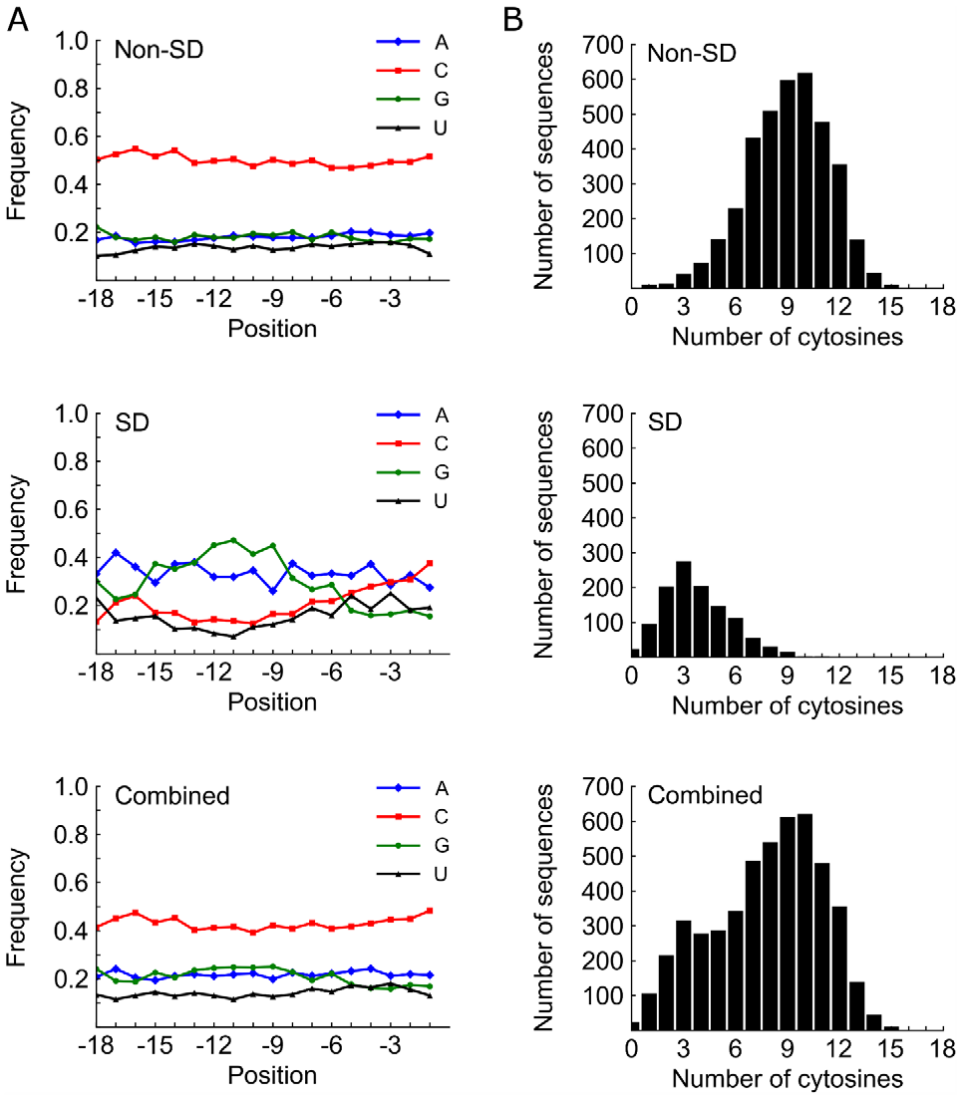
Base content versus position and prevalence of cytosine after third round of selection. (A) Base content versus position for non-SD, SD, and all sequences is shown. In the non-SD group, the cytosine content is high at all positions. In the SD group, a high frequency of guanine is detected approximately between positions −12 and −9. (B) Histograms of cytosine counts in the randomized region for non-SD, SD, and all sequences are shown.

Remarkably, of the sequences analyzed from the third round, 3,696 (76%) were considered non-SD candidates (3,491 unique). While we expected that perhaps a significant portion of these non-SD candidates could still be acting by slightly mismatched SD-anti-SD interactions, this did not appear to be the case. In fact, we observed that these sequences were highly C-rich. Of the non-SD candidates, 2,244 (61%) contained nine or more cytosines out of 18. This cytosine richness did not appear to be position-dependent. Base frequency versus position and cytosine content histograms are shown in Figure 2A and 2B, respectively, for non-SD, SD, and combined populations from the third round of selection.

### C-rich RBSs exhibit striking complementarity to the 16S rRNA

**Figure 3:**
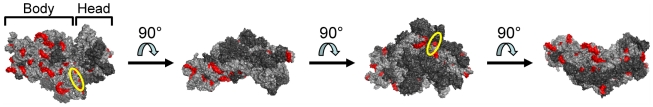
Distribution of potential sites for base-pairing of C-rich RBSs to 16S rRNA. Regions on the *E. coli* 30S ribosomal subunit with significant complementarity to the 30-5-3 library (*p*-value<0.01; Bonferroni-corrected) were determined. Significant seven-base windows that shared six bases with at least one neighboring significant window are highlighted in red (PyMOL rendering of PDB 3DF1). Four different views are shown to convey the general distribution of these potential base-pairing sites over the small ribosomal subunit. The first view shows the face that becomes buried after assembly with the large ribosomal subunit. The yellow ellipse indicates the approximate position of the anti-SD sequence. 16S rRNA = light gray; ribosomal proteins = dark gray.

We hypothesized that these C-rich sequences might be operating by base-pairing with the 16S rRNA in the 30S ribosomal subunit, which is generally G-rich. Indeed, this idea has been suggested in both prokaryotic [33] and eukaryotic [34] systems, although consensus on the issue is lacking [35], [36]. We looked at four-, five-, six-, seven-, and eight-base potential complementarities. Overlapping windows of these lengths from the 18-base randomized region of third-round products were compared to all identically-sized windows of *E. coli* 16S rRNA. We considered all 4,863 18-base regions in this analysis, including both SD and non-SD sequences. The frequency of motifs in our data set that were Watson-Crick (A/U or C/G) reverse complements of each window on the 16S rRNA was determined. We assigned a *p*-value to each window on the 16S rRNA based on the probability distribution obtained from analyzing ∼100,000 randomly generated libraries equal in size to the dataset (probability of each base = 0.25). The 30S ribosomal subunit of *E. coli* (PDB 3DF1; [37]) is shown in Figure 3 with potential mRNA-rRNA base-pairing sites shown in red. To be highly stringent, only significant (*p*<0.01; Bonferroni-corrected) seven-base windows that shared six bases with at least one neighboring significant window were highlighted. Potential mRNA-rRNA base-pairing sites primarily fell on the body of the 30S subunit on the face that becomes buried after assembly with the 50S (Figure 3, first panel). The mRNA tunnel lies between the body and head on this face. Full results from the 16S rRNA comparison are presented in Table S1. We also found that the overall propensity of the enriched library to form secondary structure resembled that of the starting library (Figure S3), underscoring the importance of primary structure (i.e., nucleotide sequence) in ribosome binding. The lack of a strong pressure for low secondary structure in the RBS region may have resulted from compensatory low secondary structure in the first ∼40 nucleotides of the coding region.

### Many C-rich motifs revealed by naïve motif search of selected RBSs

Based on the observed C-rich trend and the complementarity to the G-rich 16S rRNA, we decided to perform a naïve motif search to reveal any interesting local patterns. We determined the frequency of all possible four-, five-, six-, seven-, and eight-base motifs within the 18 bases, independent of the 16S rRNA, and asked whether specific motifs were significantly overrepresented compared to what would be expected in the naïve library (i.e., N_18_). We considered all 4,863 18-base regions from the third-round products in this analysis, including both SD and non-SD sequences. As expected based on overall base frequencies, nearly all of the top sequences were highly C-rich. More striking was that the most frequent motifs from the motif search exhibited unexpected similarities to the Kozak consensus sequence found in vertebrates. To investigate these observed similarities in more detail, the most frequent motifs found in the 18 nucleotides prior to the start codon in human (NCBI TaxID 9606) from the Transterm database [38] were considered. Four of the top nine five-base motifs in our selected sequences were also present within the top 17 motifs in human: CCACC, CCGCC, CCCGC, and GCCCC (Table 1). The full results from this motif search are provided in Table S2.

**Table 1:** Similarity of 5′ UTR motifs from selection to those from human.

5′ UTR motifs selected for fast translation	Frequency	5′ UTR motifs (18b prior to AUG) in human	Frequency
**CCACC**	0.152	**CCGCC**	0.079
**CCGCC**	0.144	GCCGC	0.071
CGCCC	0.143	GCGGC	0.067
CCCAC	0.140	CAGCC	0.062
CCCCC	0.133	GCAGC	0.061
CACCC	0.130	GGCGG	0.059
**CCCGC**	0.129	CCCAG	0.058
CCCUC	0.114	CCAGC	0.055
**GCCCC**	0.111	CGCCG	0.053
CCUCC	0.105	CGGCG	0.052
CCCCA	0.096	**CCCGC**	0.050
CCCCG	0.096	CGGCC	0.050
ACCCC	0.095	CCAGG	0.049
CUCCC	0.091	**CCACC**	0.048
CCCCU	0.084	CCGGC	0.047
**GGAGG**	0.076	GCGCC	0.047
CACGC	0.074	**GCCCC**	0.046
CACAC	0.073	CUGCC	0.046
AGGAG	0.072	CCCGG	0.046
CGCAC	0.068	**GGAGG**	0.044

5′ UTRs selected for fast-translation in an *E. coli*-based translation system exhibit striking similarity to the same region (18 bases prior to AUG) in human. The top 20 five-base motifs from each category are shown. Five particular motifs (bold) were present in both sets. Similar results were obtained for other motif lengths.

Previous studies involving prokaryotic RBSs have not recognized the inherent ability of 70S ribosomes to efficiently translate from C-rich start sequences, including those resembling the Kozak consensus sequence, probably because those studies were not conducted in a minimal translation system. The Kozak sequence has been previously investigated for its complementarity to the rRNA of the small subunit in eukaryotes [39], much as we have done with our selected RBS sequences. The Discussion provides further insight into the parallels between our study and this previous analysis performed in a eukaryotic system, suggesting universal features of the ribosome.

### SD function is enhanced by AC dinucleotide repeats

All motifs found to be significant in the motif search (FDR<0.01) were given further consideration for their co-occurrence with other significant motifs within the same 18-base randomized RBS region. A co-occurrence metric was defined as the number of RBS regions that contained both motif 1 and motif 2 divided by the number of RBS regions that contained motif 2 only. Through this measure, we identified “enhancers” of canonical SD motifs. Variations of an AC dinucleotide repeat were found to correlate strongly with GGAGG. Interestingly, AC dinucleotide repeats downstream of the start codon have previously been reported to enhance translation [40]. Results from the co-occurrence analysis are provided in Table S3 for all pairs of significant motifs that had a non-zero co-occurrence metric. Co-occurrence of C-rich motifs with other C-rich motifs is also evident in Table S3.

### Poly-cytosine alone is not sufficient to promote fast translation

**Figure 4:**
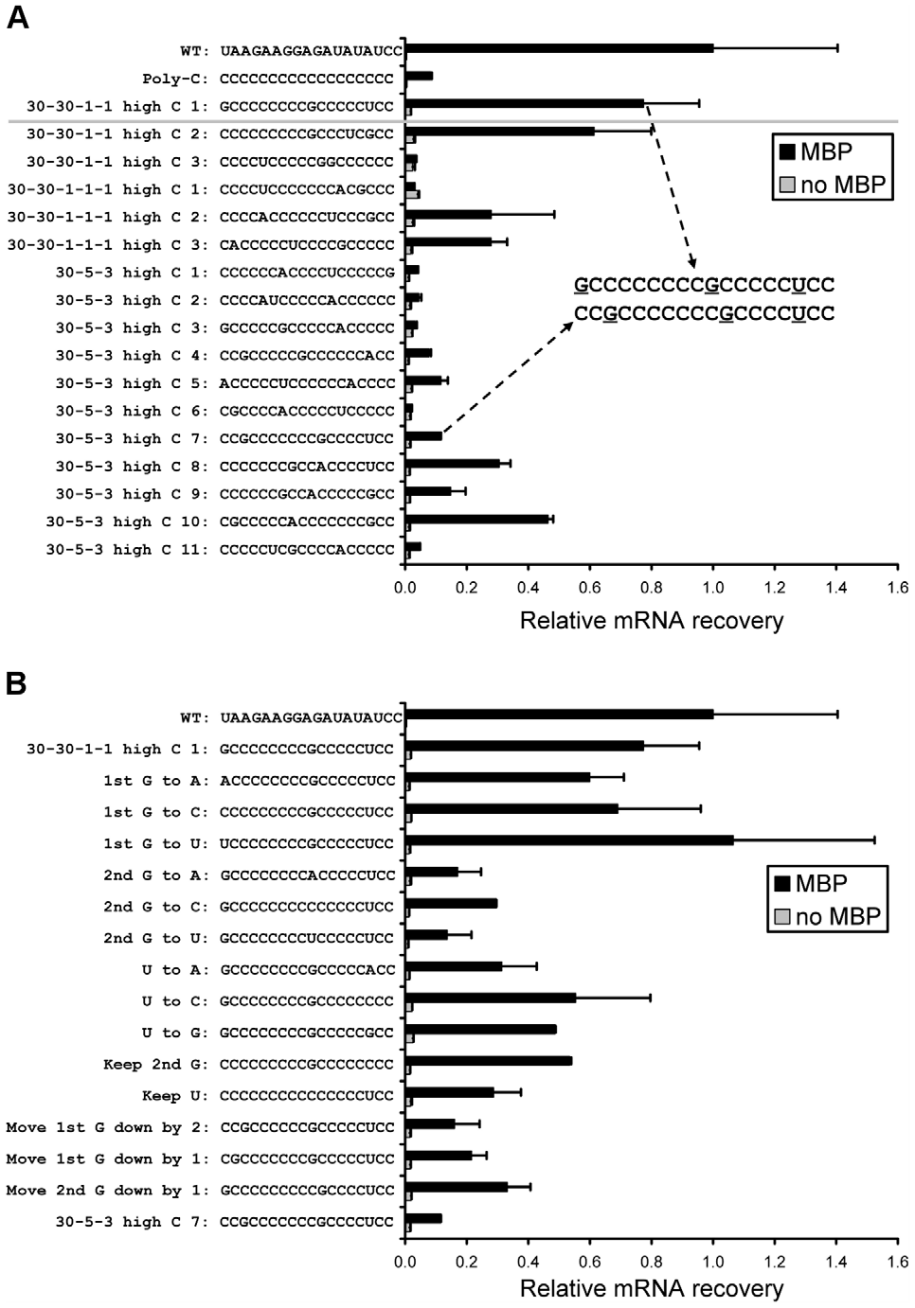
Single-clone ribosome display. (A) The poly-C consensus sequence displayed very low activity relative to the WT pRDV sequence, but one of the most C-rich clones had an efficiency of ∼80% compared to WT (cf. the three sequences above the horizontal gray line). Nearly all highly C-rich clones exhibited activity. Interestingly, the most efficient clones (30-30-1-1 high C 1 and 30-30-1-1 high C 2) both contained G at position -9 and a non-C nucleotide at position −3. Clone 30-5-3 high C 7 was highly similar to 30-30-1-1 high C 1, although the latter was far more efficient. (B) A panel of mutants was created to study the differential activity of two similar clones in (A). Mutational analysis revealed the importance of positions −9 and −3. Error bars indicate half range of duplicates. MBP = maltose-binding protein.

We tested the poly-C consensus RBS against the WT pRDV RBS and one of our C-rich RBS clones in single-clone ribosome display. mRNA recovery was quantified by qRT-PCR (Figure 4A, top three sequences). Surprisingly, the poly-C consensus was not efficient. To determine which non-C nucleotides in a C-rich context enabled efficient translation, we performed single-clone ribosome display on a panel of our most C-rich clones (with cytosines at 15 of 18 positions). We considered clones from the basic selection scheme (three rounds: 30 min, 5 min, 3 min translation; “30-5-3”) as well as two alternate selection schemes (four rounds: 30 min, 30 min, 1 min, 1 min translation with or without an additional 1-min round; “30-30-1-1-1” and “30-30-1-1,” respectively). mRNA recovery from the alternate selection schemes, quantified by qRT-PCR, is presented in Figure S1. Most clones exhibited activity well above background (Figure 4A); however, highly similar clones exhibited greatly different activities, suggesting that the placement of non-C nucleotides in a C-rich context is crucial. We investigated two clones, 30-30-1-1 high C 1 ( GCCCCCCCCGCCCCCUCC; ∼80% WT activity) and 30-5-3 high C 7 (CCGCCCCCCCGCCCCUCC; ∼10% WT activity) more closely. These two clones differ only in the position of two guanines: one near the 5′ end of the random region and one near the middle. To investigate the nucleotides responsible for the differential activity of these two clones, we performed single-clone ribosome display on an extended panel of mutant RBSs (Figure 4B). Mutation of the first G to A, C, or U in 30-30-1-1 high C 1 had no major effect, while mutation of the second G to A, C, or U greatly decreased activity. Mutation of the U to A, C, or G also decreased activity. Finally, shifting the first G from −18 to −17 or −16 or shifting the second G from −9 to −8 greatly decreased activity.

### Most efficient highly C-rich RBS is sensitive to oligonucleotide competition

**Figure 5:**
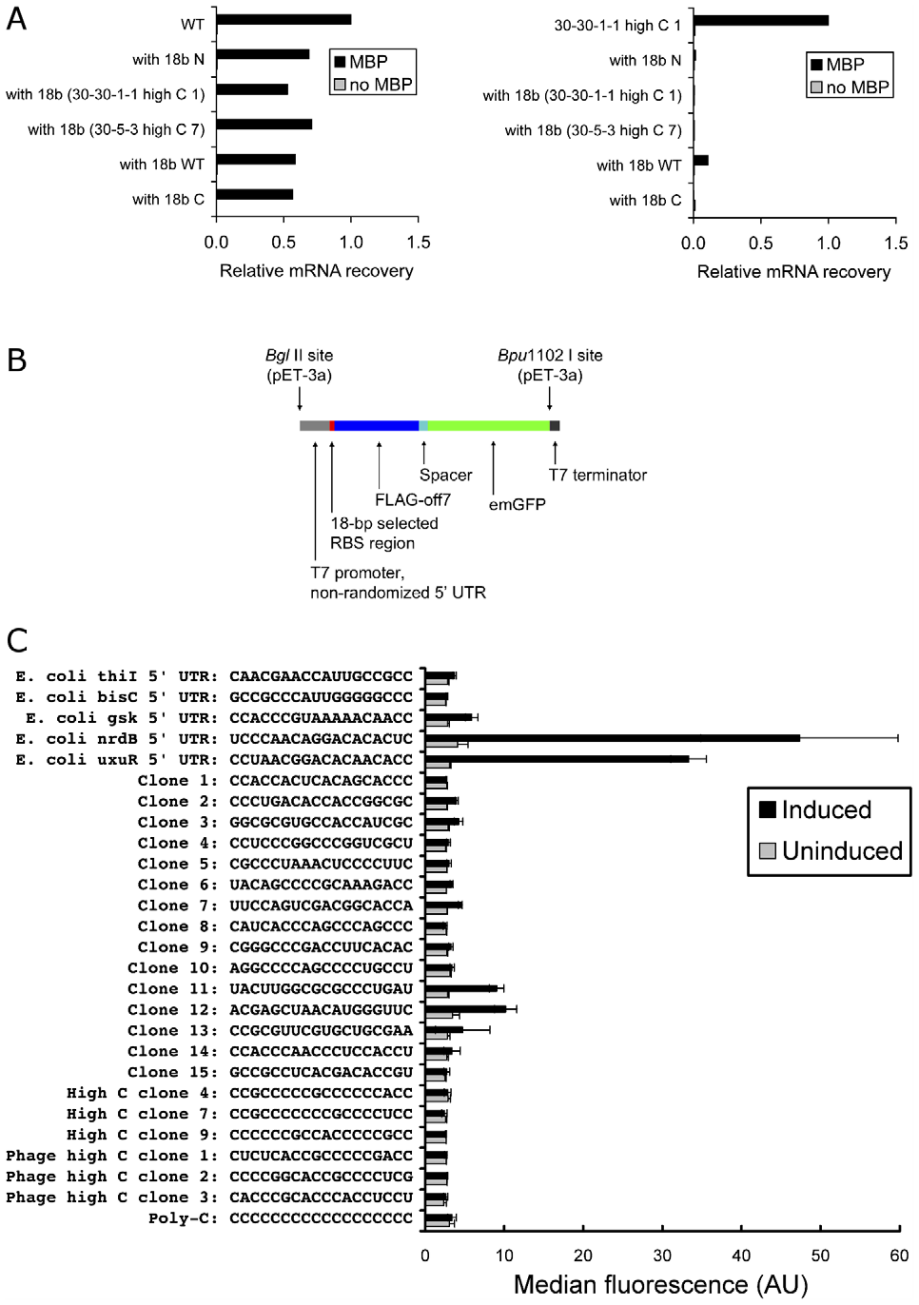
*In vitro* competition and *in vivo* expression. (A) WT and Clone 30-30-1-1 high C 1 were differentially affected by 400 µM 18-base ssDNA oligonucleotide competitors: random (N), clone 30-30-1-1 high C 1, a similar C-rich clone (30-5-3 high C 7), WT, and poly-C. MBP = maltose-binding protein. (B) Expression cassettes containing an RBS followed by FLAG-off7-emGFP were built by assembly PCR and cloned into pET-3a, which was used to transform BL21(DE3)pLysS. (C) Green fluorescence (excitation/emission: 487/509 nm) was quantified by flow cytometry after 4 h induction with 1 mM IPTG. The average median fluorescence of induced and uninduced clones is shown. Error bars represent standard deviation of at least three experiments. The first five sequences are the *E. coli* 5′ UTRs (18 bases before the start codon) having the most similarity to individual selected library members. They also happen to be highly C-rich for *E. coli*. Of these, only the *E. coli nrdB* 5′ UTR (UCCCAACAGGACACACUC) contains an SD motif (underlined). The next 15 sequences (“Clone 1” to “Clone 15”) are the most prevalent clones sequenced from the 30-5-3 selection scheme. The next six sequences are three of the most C-rich clones sequenced and three of the most C-rich 5′ UTRs present in phages from the EMBL-EBI database (*Burkholderia* phage KS14: HM461982; *Mycobacterium* phage Nigel: EU770221; and *Synechococcus* phage Syn5: EF372997, respectively). The final sequence is poly-C, which does not perform well. WT average median fluorescence (not shown) was extremely high (1417±178 AU induced, 15.2±15.6 AU uninduced).

To investigate our base-pairing hypothesis experimentally, we performed single-clone ribosome display of WT and a C-rich clone (30-30-1-1 high C 1) in the presence of various ssDNA oligonucleotide competitors. We used five different 18-base competitors: random (N), clone 30-30-1-1 high C 1, a similar C-rich clone (30-5-3 high C 7), WT, and poly-C. This panel of competitors was designed to interrogate specificity of translational inhibition (if any). The activity of the WT clone was only moderately inhibited by a large excess of any oligonucleotide, while the activity of the C-rich clone was effectively eliminated by random or C-rich competitors. Even WT competitor strongly inhibited the C-rich clone, though to a lesser extent than the other competitors (Figure 5A).

### C-rich RBSs are not efficient in *E. coli*

Finally, we tested a panel of clones *in vivo* by fusing off7 to emGFP through a short linker (Figure 5B) and then monitoring green fluorescence in *E. coli* (Figure 5C). This panel of clones included five C-rich pre-AUG 18-base regions from *E. coli* (derived from the 5′ UTRs of *thiI*, *bisC*, *gsk*, *nrdB*, and *uxuR*), 15 clones from the 30-5-3 selection with maximal redundancy (two with four instances, 13 with three instances), three representative clones with high C content from the 30-5-3 selection, three of the most C-rich 18-base upstream regions present in phage annotated on EMBL-EBI, and the WT pRDV sequence. The average median fluorescence of these 31 clones from at least three independent experiments is provided in Figure 5C. The induced WT signal was over 580 times above that of 30-5-3 high C 7, while 5′ UTR mRNA levels were only about 14-fold different, which only accounts for a small fraction of the discrepancy in protein levels. This suggests that observed differences in the *in vivo* responses for WT and the C-rich clones can be primarily attributed to their differential translational efficiencies. The poor performance of C-rich upstream regions from phage was not unexpected, because the phage from which those 5′ UTRs were derived do not naturally infect *E. coli*. In support of a base-pairing mechanism, native hosts of phage having C-rich 5′ UTRs (e.g., *Burkholderia cenocepacia*, *Mycobacterium tuberculosis* H37Rv, and *Synechococcus* sp. WH 8109) clearly have more C-rich 5′ UTR profiles than *E. coli* (Figure S4). Although most of our selected clones performed poorly *in vivo*, at least two synthetic sequences (30-5-3 clones 11 and 12) exhibited activity >2-fold over background, on par with that of the native 18-base sequence immediately upstream of *E. coli gsk*. In light of our competition experiments *in vitro*, we conclude that the *in vivo* environment of *E. coli* contains a large quantity of endogenous RNA species that out-competes mRNA containing a C-rich RBS. However, given the two examples of synthetic sequences that retain some activity *in vivo*, the magnitude of this competition effect is likely to be sequence-specific.

## Discussion

### Ribosome display as a discovery tool

Ribosome display, employed as a tool for investigating the non-coding regions of mRNA, particularly in a minimal translation system, has the potential to generate insights not available through previous studies. The large library sizes of ribosome display (easily up to ∼10^14^ with reasonable scale-up) allow much more exhaustive sampling than any technique requiring a transformation step. Coupling these selections with high-throughput sequencing enables the discovery of statistically relevant motifs in the selected sequences. Furthermore, a synthetic biology approach, in which a well-defined translation system is used, can elucidate inherent capabilities of the translational machinery and new insights into the function of natural biomolecules that may be difficult to uncover in a native biological context. In the present study, ribosome display and high-throughput sequencing were used to demonstrate that efficient translation in a minimal, well-defined, *E. coli-*based *in vitro* translation system can be mediated by C-rich RBSs which are postulated to base-pair to G-rich 16S rRNA motifs.

The identification of highly C-rich RBSs using ribosome display in the PURExpress system underscores the high structural and functional conservation of the ribosome and shows that, if given optimal conditions, ribosomes from one species can bind to mRNAs which are more frequent in other species in nature. Highly C-rich RBSs have been found in multiple diverse organisms, including non-*E. coli* phage, lower eukaryotes, plants, and vertebrates. A discussion of such natural examples as well as the notable lack of C-rich RBSs in *E. coli* genes is presented further below.

### C-rich local consensus sequences

Interestingly, our selected sequences had an overall consensus of poly-C, although the poly-C sequence by itself was not efficient. The inability of this global consensus sequence to promote efficient translation in the PURExpress system provided an important insight for this study: the overall 18-base consensus does not describe the selected library well. Instead, shorter, significant (FDR<0.01) motifs that were analyzed independently of the 16S rRNA comprise many local consensus sequences. There was no striking position-dependence of individual local consensus sequences when viewed over the entire population; this contrasted starkly with the SD motifs, which were much more position-dependent.

Additionally, our consensus did not contain a “purine peak” at position -3, which is frequently found in humans and other vertebrates [6]. This purine peak may not be present in lower eukaryotes such as *Encephalitozoon cuniculi*, an intracellular eukaryotic parasite that frequently infects immunodeficient patients. This organism has short leaders but also contains a poly-C consensus prior to the start codon [24], much as we observed in our selections. The mechanism by which this parasite initiates translation is currently unknown, although the present study may provide some insight by demonstrating non-native functions of *E. coli* ribosomes that reflect the RBS preferences of other organisms.

### Presence of C-rich sequences in non–*E. coli* phage

The presence of C-rich sequences in phage 5′ UTRs suggests that some aspect of the host environment enables their fast translation. Based on our observations of the effect of competitor oligonucleotides, we propose that phage with C-rich 5′ UTRs best utilize these genes in an environment low in nucleic acids. Interestingly, the *Burkholderia* phage KS14 contains its most C-rich 5′ UTR prior to its gene for tail completion protein R. Therefore, at least one of the most C-rich motifs in phage precedes a highly-produced late protein (i.e., structural protein), although the general lack of annotation of phage genes limits our analysis. In late-stage infection, host mRNAs are often repressed, globally or locally [41]–[43], so highly efficient C-rich RBSs may also serve to temporally control the production of certain proteins (e.g., structural proteins should be abundantly synthesized, but only towards the end of phage assembly). Phage with C-rich 5′ UTRs may infect slow-growing organisms, such as *M. tuberculosis* [44], which may have lower basal mRNA content than other species, such as *E. coli*.

### Support for multiple-contact model

The co-occurrence of multiple short C-rich motifs within the 18-base RBS region suggests that multiple segments of the RBS may interact either sequentially or concurrently with the 16S rRNA, which has multiple binding sites itself. Fast binding and unbinding of these short mRNA motifs to various positions on the ribosome may help maintain a high concentration of ribosomes near the start codon while still permitting necessary mRNA repositioning for initiation and transition to elongation. The concept of multiple mRNA-rRNA interactions has been described as clustering for eukaryotic ribosomes [45], and we suggest that a similar mechanism may be at work here. In theory, the entire length of an mRNA molecule may be able to interact with the rRNA, but it is the initiation region that determines the accessibility of the start codon and the efficiency of forming the preinitiation complex [46].

### Further evidence of base-pairing in plants

mRNA-rRNA complementarity has also been found to enhance translation in plants. For example, the ARC-1 element (18S rRNA positions 1115–1124, GGGGGAGUAU) was shown to enhance translation when present in the leader or intercistronic region of model mRNAs [22]. This study also showed that linking three or more copies of this enhancer element augmented translation to levels directed by natural enhancers in tobacco mosaic virus and potato virus Y mRNAs. A subsequent investigation by the same group showed that enhancer activity was inhibited in the presence of competitor oligonucleotide and that the same oligonucleotide, when modified at the 5′ end with an alkylating group, hybridized to the ARC-1 element [23]. Intriguingly, part of the homologous *E. coli* 16S rRNA region was found to be a potential mRNA hybridization site in our study.

### Universality of ribosome binding sites

While it has been recognized for some time that the ribosome is, in fact, a broad-specificity ribozyme, there has not been much discussion of universally efficient RBSs in the literature. Recently, species-independent translational sequences have been reported [47]. These utilize a poly-A or UUUUA repeat to create a long, unstructured region prior to the start codon. The impressive efficiency of poly-A and (to a lesser extent) poly-U RBS constructs *in vitro* and *in vivo* is consistent with this report (Figure S5). An analysis of all eukaryotic start sequences has identified two distinct patterns, AAAAAA and GCCGCC, which supposedly work by distinct mechanisms [48]. *S. cerevisiae*, for example, prefers the former consensus, while human and other vertebrates generally use a sequence closer to the latter. Interestingly, the *S. cerevisiae* rRNA is rich in poly-U tracts, while vertebrate rRNAs are generally rich in poly-G tracts, further supporting the notion that transient rRNA-mRNA base-pairing may be a broad-specificity mechanism for translational regulation. Additionally, the base-pairing of Kozak sequences to the 18S rRNA has been proposed [39]. In this study, Sarge and Maxwell presented a competitive-displacement model for the initiation of translation involving the intermolecular base-pairing of 5S rRNA, 18S rRNA, and mRNA. They proposed that a particular segment of the 18S rRNA complementary to the Kozak sequence was able to lock the mRNA in place so that a 48S preinitiation complex could form. The 60S subunit would then join, and the 5S rRNA would displace the mRNA. Although the details of this model may not apply directly to the present study, there is indeed precedence in the literature for C-rich, Kozak-like sequences to show evidence of binding to the rRNA of the small subunit prior to initiation of translation [39]. More generally, the fact that ribosomes from distantly related organisms (i.e., *E. coli* and human) can use both poly-A and Kozak-like patterns to initiate translation provides interesting material for further research on the universality of the ribosome.

### Experimental and computational assumptions and justifications

Because *E. coli* grows quickly and has large amounts of RNA compared to slower-growing bacteria, it is quite possible that competition for potential pairing sites on the ribosome from other nucleic acids or other molecules prevents translation of mRNAs containing C-rich RBSs. We make this assertion based on the fact that C-rich sequences are inhibited from facilitating translation *in vitro* when competitor oligonucleotides are added. Most *E. coli* genes are not C-rich, which highlights the fact that our results using *E. coli* ribosomes must be considered in the context in which they were selected. Our objective was to gain insight into the inherent capabilities of the ribosome, so we used a minimal *in vitro* translation system; by contrast, if the ultimate goal of a study is to simply increase *in vivo* expression, the selections should be performed *in vivo*. It is theoretically possible that C-rich mRNA sequences may have been selected in part because of their ability to outcompete other sequences for binding to ribosomes, not necessarily because they are the most efficient at promoting fast translation, which requires speed in forming the initiation complex and also in transitioning to elongation. However, the enriched libraries performed translation very well overall, suggesting that this should not be a major concern.

The computational analysis was performed without knowledge-based bias of where base-pairing occurs in available ribosomal crystal structures. Many of the potential pairing sites are at least partially base-paired in the crystal structure, but a large number of these sites may be vulnerable to displacement at the translation temperature. The ribosome is a highly dynamic macromolecule and surface-proximal potential pairing sites could easily be involved in transient complementary interactions.

Additionally, it is possible that the 23S and/or 5S rRNAs of the large ribosomal subunit may be involved in some of the interactions. The ribosomes in the PURExpress system are 70S complexes, although IF3 is able to separate them [49]. When an analysis identical to that shown in Figure 3 was performed with the 23S rRNA and 5S rRNA, we found 56 and 2 potential pairing sites, respectively. Based on what is known about the translation of leadered mRNAs, we would expect the 16S rRNA to play the major role; however, we cannot exclude the possibility of the large subunit rRNAs mediating mRNA-ribosome interactions, which, for example, could serve to increase the local mRNA concentration until a binding event resulting in translation initiation occurred.

Finally, based on the traditional model of prokaryotic translation, we assume that the 18-base randomized region before the coding region functions primarily in translation initiation, although it is possible that this region could exert some effects on elongation, perhaps if the C-rich sequences could interact with the ribosome in or near the exit tunnel to facilitate mRNA movement through the 70S ribosome. Differences in mRNA recovery could theoretically result from effects of the randomized RBS region on elongation, but current dogma suggests that this is less likely.

### Inherent capabilities of the ribosome narrowed by *in vivo* conditions

**Figure 6:**
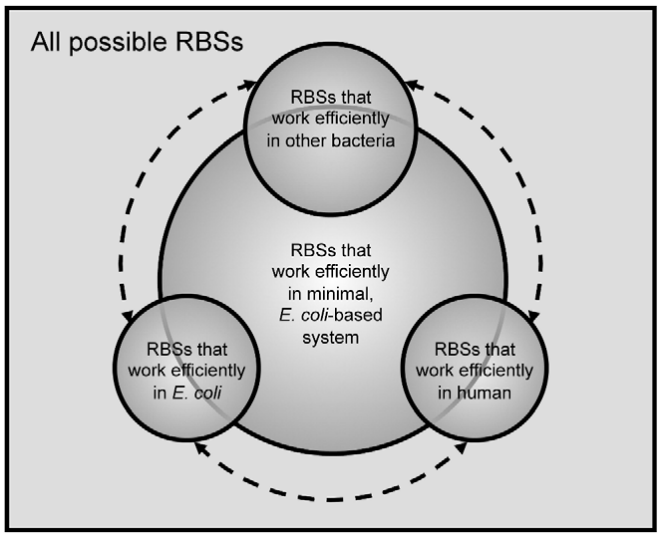
Model for RBS functioning *in vitro* and *in vivo*. Of all possible RBSs, a certain subset works efficiently in a minimal, *E. coli-*based system. Of these, some RBSs work efficiently in *E. coli* (e.g., WT pRDV RBS), in other bacteria, and in distantly-related organisms, such as human, which contains many C-rich motifs near the start codon. It is likely that these three groups have some overlap (represented by dashed lines), but for the purposes of making generalizations, they have been drawn separately. Finally, certain RBSs that work efficiently in *E. coli* most likely require *in vivo* factors not present in the minimal system to function efficiently; the same can be said of certain RBSs that work efficiently in other bacteria or in human. Moreover, changing the context of an RBS may greatly change its efficiency and move it into a different space in the diagram. Nevertheless, broad-specificity mRNA-rRNA base-pairing suggested by our study using a minimal *E. coli*-based system may serve as a unifying mechanism for the functioning of a subset of RBSs from diverse hosts.

In the present study, we uncovered both expected SD sequences and unexpected C-rich non-SD sequences as efficient RBSs in a minimal, reconstituted *E. coli* system. All of these sequences appear to operate by base-pairing to the rRNA of the small subunit of the ribosome. This general design principle represents an inherent, broad-specificity mechanism for efficient translation *in vitro* that is further refined *in vivo* (Figure 6). Notably, the specific subset of RBSs that are utilized *in vivo* can be different for different hosts: *E. coli* does not appear to utilize C-rich RBSs in translating its native genes, likely due to the fact that SD sequences perform more robustly in its intracellular environment; bacteria such as *Mycobacterium tuberculosis* have more C-rich 5′ UTRs than *E. coli*, suggesting that both SD and C-rich RBSs play functional roles in these hosts; and human and other vertebrates widely use C-rich sequences (including Kozak-like motifs), but not SD-like sequences, for translation. Our results suggest the intriguing possibility that RBSs in different organisms that may appear unrelated by sequence may actually share a common mechanism for translation initiation based on broad-specificity mRNA-rRNA base-pairing.

## Materials and Methods

### Library construction and cloning for single-clone studies

Procedures for construction of the naïve RBS library, the single-clone constructs used for single-clone ribosome display, and the single-clone constructs used for the *in vivo* expression studies are provided in Text S1. All oligonucleotides specific to these procedures are listed in Table S4.

### Ribosome display

Ribosome display selection particles were generated using the well-defined PURExpress *in vitro* protein synthesis kit (New England Biolabs). Since the concentration of ribosomes in the standard PURExpress reaction is specified by the manufacturer (2.4 µM), we could accurately control the RNA∶ribosome ratio (∼10∶1 in the first round, ∼4∶1 in subsequent rounds) by using RNA, and not DNA, as the template. Kit components (Solution A and Solution B), RNA, RNasin ribonuclease inhibitor (Promega, Madison, WI) and water (if necessary for dilution) were mixed according to the manufacturer's instructions, except in cases where fewer ribosomes (found in Solution B) were required to achieve high RNA∶ribosome ratios. In the first round of selection, 18 µg mRNA (corresponding to ∼3.7×10^13^ molecules) was used in a total volume of 16 µL. The translation reaction was incubated at 37°C for 30 min in order to allow full translation of any mRNAs that contained an RBS. The translation was stopped using 400 µL cold WB buffer (50 mM Tris-acetate, pH 7.5 at 4°C, 150 mM NaCl, 50 mM magnesium acetate; [28]). Then, the stopped translation was subjected to ultrafiltration using a 100 kDa cut-off Microcon centrifugal filter unit (Millipore, Billerica, MA). The ultrafiltered translation was diluted up to 100 µL with WBT (WB plus 0.05% Tween-20) containing RNasin, mixed thoroughly, and used for binding in one well. Binding was performed using NUNC Maxisorp plates (Thermo Fisher Scientific, Rochester, NY) prepared as follows: plates were coated with 100 µL 66 nM NeutrAvidin (Thermo Fisher Scientific) for at least 16 h at 4°C, washed with TBS (50 mM Tris-HCl, pH 7.4 at 4°C, 150 mM NaCl), blocked with 25 mg/mL casein (Sigma-Aldrich, St. Louis, MO) or 10 mg/mL BlockAce (AbD Serotec, Raleigh, NC) in TBS at room temperature for at least 1 h with shaking, incubated with biotinylated maltose-binding protein of *E. coli* in blocking solution for at least 1 h at 4°C with shaking, and washed with TBS and WBT. Binding was performed for 1 h at 4°C with shaking. The plate was washed with WBT and then once with WB prior to reverse transcription.

Reverse transcription was performed using AffinityScript reverse transcriptase (Agilent Technologies, Santa Clara, CA) and reverse primer tolA_stops_HindIII_rev (5′-GGC CAC CAG ATC CAA GCT T-3′) that anneals just downstream of off7. An *in situ* reverse transcription protocol [50] was adapted as follows: 12 µL Solution 1 (11.375 µL water and 0.125 µL reverse primer tolA_stops_HindIII_rev) was pipetted into the well, incubated at 70°C for 10 min, and removed from heat for 5 min. 8 µL Solution 2 (3 µL dNTPs [5 mM each], 2 µL 10× AffinityScript buffer, 2 µL 0.1 M DTT, and 1 µL AffinityScript reverse transcriptase) was added and the reaction was incubated at 45°C for 1 h, then heat-inactivated at 70°C for 15 min. Half of the 20 µL reaction was taken as template for a 100 µL PCR with primers T7_ext_fwd (5′-ATA CGA AAT TAA TAC GAC TCA CTA TAG GGA CAC CAC AAC GGT TTC CCT AAT TGT GAG CGG ATA ACA ATA GAA ATA ATT TTG TTT AAC TT-3′) and tolA_stops_HindIII_rev. T7_ext_fwd anneals just before the 18-base randomized region to maximize recovery; additionally, by only recovering those sequences which contain enough bases upstream of the RBS region to facilitate primer annealing, we can be assured that potential nuclease processing near or within the RBS is not significantly influencing our results. The PCR product (624 bp) was gel-purified and digested with HindIII. The tolA spacer was made by amplifying pRDVstops-off7 with HindIII_tolA_stops_fwd (5′-TAC TGC AAC AAG CTT GGA TCT GGT GGC CAG AA-3′) and tolAk (5′-CCG CAC ACC AGT AAG GTG TGC GGT TTC AGT TGC CGC TTT CTT TCT-3′) [30] to form a 303 bp product. Both pieces were digested with HindIII, ligated, and gel-purified to generate the full-length construct (899 bp). This product was amplified with T7_no_BsaI (5′-ATA CGA AAT TAA TAC GAC TCA CTA TAG GGA CAC CAC AAC GG-3′) and tolAk to obtain enough product for transcription for the second round.

Different selection schemes were performed based on this first round with 30 min translation. In one scheme, two additional rounds (5 min and 3 min, respectively) were performed with no ultrafiltration (“30-5-3” selection). In an alternate scheme, three additional rounds (30 min, 1 min, and 1 min) were performed with ultrafiltration (“30-30-1-1” selection) followed by a final 1-min round without ultrafiltration (“30-30-1-1-1” selection). The volume in round 1 (16 µL) was chosen to be higher than in subsequent rounds because we expected few mRNAs in the original library to contain a functional RBS. After the initial round, the pool was highly enriched, so much smaller volumes could be used effectively. Pipetting errors were kept to a minimum by preparing translation reactions of at least 5 µL. After translation, the reactions were diluted, divided into four parts (each containing at least 1.25 µL translation), and used for binding in duplicate positive wells and duplicate negative wells. Thin-walled PCR tubes were used for incubation, so all volumes quickly reached the translation temperature (37°C). The products of all rounds were quantified by qRT-PCR on the Applied Biosystems 7300 Real-Time PCR System using TaqMan Universal PCR Master Mix (Applied Biosystems), off7_fwd (5′-TCC ATC GAC AAC GGT AAC GA-3′), tolA_stops_HindIII_rev, and off7_probe (6-FAM-5′-TGG CTG AAA TCC TG-3′). Products from all selection schemes were sequenced on a Roche/454 GS FLX sequencer at the University of Pennsylvania DNA Sequencing Facility. Sanger sequencing was also performed on the 30-30-1-1 selection. Sequences from the 30-5-3 selection were chosen for extensive sequence analysis. Highly C-rich clones from the 30-30-1-1 and 30-30-1-1-1 selections were also investigated. Prior to some rounds (5 min and 3 min rounds from 30-5-3 selection and final 1 min round from 30-30-1-1-1 selection), off7-tolA amplified with BsaI_FLAG_fwd2 (5′-ACT GAT TAG GTC TCA GAT GAC GAT GAC AAA GGA TC-3′) and tolAk was digested with BsaI and ligated onto the BsaI-digested library, made by PCR on the reverse transcription product using BsaI_FLAG_rev (5′-ACT GAT TAG GTC TCT CAT CTT TGT AGT CCG CCA T-3′) and T7_no_BsaI.

### Single-clone ribosome display

Sequence-verified minipreps were amplified with T7_no_BsaI and tolAk for *in vitro* transcription. Generally, ∼1 µL translation was used per well to make sure that the signal stayed in the linear range. The RNA∶ribosome ratio was 4∶1 in all experiments. Translation was performed for 10 min, which is optimal for WT. If applicable, DNA oligonucleotide at a concentration of 2.5 mM was added to the translation to a final concentration of ∼400 µM, which provided ∼40-fold molar excess compared to mRNA (∼9.6 µM). Five different DNA oligonucleotides were used: 18b_N, 5′-NNN NNN NNN NNN NNN NNN-3′; 18b_(30-30-1-1_high_C_clone_1), 5′-GCC CCC CCC GCC CCC TCC-3′; 18b_(30-5-3_high_C_clone_7), 5′-CCG CCC CCC CGC CCC TCC-3′; 18b_WT, 5′-TAA GAA GGA GAT ATA TCC-3′; and 18b_C, 5′-CCC CCC CCC CCC CCC CCC-3′. Oligonucleotides were added to the translation just prior to the mRNA.

### *In vivo* experiments

Selected sequences were cloned into pET-3a (Novagen, Madison, WI) and sequence-verified minipreps were transformed into *E. coli* BL21(DE3)pLysS (Agilent, Santa Clara, CA) for expression. Individual colonies were inoculated into LB containing 100 µg/mL ampicillin (to maintain pET-3a) and 50 µg/mL chloramphenicol (to maintain pLysS) and grown for ∼16 h overnight at 37°C. Ampicillin was omitted from the negative control (background strain). The next morning, cultures were diluted 1∶50 in 1 mL LB without antibiotic and allowed to grow for 3 h at 37°C. Half of each culture was then induced with 1 mM isopropyl β-D-1-thiogalactopyranoside (IPTG). Cultures were grown for another 4 h at 37°C and analyzed on a Guava flow cytometer (Millipore). The average median fluorescence of three separate experiments was used to determine whether or not induction was appreciable (i.e., greater than two-fold over background fluorescence of the strain).

The 5′ UTRs of WT and 30-5-3 high C 7 were quantified using qRT-PCR with 5′_UTR_qPCR_fwd (5′-CCA CAA CGG TTT CCC TAA TTG T-3′), FLAG_qPCR_rev (5′-GTC ATC TTT GTA GTC CGC CAT-3′), and 5′_UTR_probe (6-FAM-5′-AGC GGA TAA CAA TAG AAA T-3′).

### Data analysis

Raw sequences were filtered to make sure the randomized region was of the expected length (18 bases) and in the expected context (TGTTTAACTT upstream and ATGGCGGACT downstream). Sequences with an in-frame ATG present in the randomized region were excluded from analysis. For the rRNA comparison, a virtual library of 4,863 random 18-base sequences was generated (equal in size to the actual sequence pool analyzed). From each 18-base sequence, 19−*k* windows of length *k* were considered for *k* = 4–8. These 4,863×(19−*k*) windows were compared to *E. coli* 16S rRNA, and the number of reverse complements present in the virtual library for each window of length *k* on the 16S rRNA was recorded. Approximately 100,000 virtual libraries of this sort were generated to develop a probability distribution at each index of the 16S rRNA starting a *k*-base window. Bonferroni-corrected *p*-values are presented as P.rand in Table S1. The significance threshold was set at 0.01. For *k* = 7, significant windows neighboring at least one other significant window were considered to be part of a group of significant windows. PyMOL [51] was used to visualize these groups on the crystal structure. There appeared to be no correlation between the position of these groups on the crystal structure and the position of the complementary motif within the randomized region. Permuted (scrambled) 5′ UTRs were also used to calculate *p*-values (Bonferroni-corrected; P.perm in Table S1). P.rand allows us to recognize sequences that deviate from randomness in terms of their base composition and order of bases, while P.perm allows us to recognize the importance of the order of bases only. For the naïve motif search, all possible *k*-base motifs, *k* = 4–8, were generated. The virtual libraries (with random or scrambled 5′ UTRs) were again generated and the incidence of each *k*-base motif was assessed; to correct for multiple tests, FDR was applied, and the resulting *q*-values for the motif search are presented as Q.rand and Q.perm in Table S2. To analyze dependencies between motifs, each significant *k*-base motif (FDR<0.01) was assessed to determine if it was more likely to occur in a 5′ UTR context containing another particular motif. This dependency was quantified by a co-occurrence metric: [# 5′ UTRs having non-overlapping motifs 1 and 2]/[# 5′ UTRs having motif 2]. These values (when non-zero) are reported in Table S3.

mRNA secondary structure analysis was performed using the following procedure, which was adapted from previously published work [52]. Sequencing reads of selected library sequences were computationally trimmed to yield mRNA molecules consisting of a 26-base region immediately prior to the randomized region, the 18-base randomized region immediately prior to the start codon, and another 26-base region starting from the start codon. Each 70-base mRNA molecule was further processed to yield five overlapping 30-base windows using an offset of 10 bases. Finally, each 30-base window was assessed for secondary structure using the UNAFold suite (program melt.pl), and the corresponding *ΔG* values were recorded. For comparison, a library of 350,000 simulated mRNA molecules having random 18-base regions (probability of each base = 0.25) was assessed for secondary structure using the procedure described above.

## Supporting Information

Figure S1 mRNA recovery. mRNA recovery was quantified by qRT-PCR after each round in (A) the basic 30-5-3 selection and (B) the alternate 30-30-1-1-1 selection. The translation time, translation volume, and ultrafiltration status are provided for each round. Where indicated, a “check” round was performed in parallel to the actual round to verify enrichment or to test a more stringent selection. In (A), the Round 3 check verified that enrichment had occurred between rounds 2 and 3. In (B), the Round 3 check verified enrichment, while the Round 4 check verified that an appropriate level of stringency had been applied. Error bars, when shown, indicate the half range of duplicate wells. The negative control (no MBP) was not performed in the first round. MBP = maltose-binding protein.
(TIF)

Figure S2 SD sequences in the 30-5-3 selection. (A) The alignment of study-defined SD motifs (red) with the 3′ tail of the 16S rRNA (black) is shown. (B) Position-dependent and overall enrichment of SD sequences over three rounds (Rd1, Rd2, Rd3) is shown. For comparison, we present all ten four-base subsets of the reverse complement (5′-UAAGGAGGUGAUC-3′) to the 13 unpaired bases at the 3′ end of the 16S rRNA (5′-GAUCACCUCCUUA-3′) in our selected sequences: UAAG, AAGG, AGGA, GGAG, GAGG, AGGU, GGUG, GUGA, UGAU, and GAUC. All SD motifs exhibited position-dependent enrichment according to their alignment with the 16S rRNA.
(TIF)

Figure S3 Histograms of *ΔG* values. Histograms of *ΔG* values in five 30-base sliding windows (offset by 10 bases) in a 70-base region centered on the 18-base randomized region in theoretical naïve (top) and selected (bottom) library from the basic selection are shown. The similarity of the distributions suggests no strong pressure for less or more secondary structure than a random library.
(TIF)

Figure S4 Histograms of natural cytosine content. Histograms of cytosine content in natural 5′ UTRs of *E. coli* K12 W3110 (NCBI TaxID: 316407) and three representative organisms that are infected by bacteriophage having very high cytosine content in at least one 5′ UTR (*Burkholderia cenocepacia*, TaxID: 331272, infected by *Burkholderia* phage KS14; *Mycobacterium tuberculosis* H37Rv, TaxID: 83332, infected by *Mycobacterium* phage Nigel; *Synechococcus* sp. WH 8109, TaxID: 166314, infected by *Synechococcus* phage Syn5) are shown. 5′ UTR datasets for all organisms except *Synechococcus* were obtained from the Transterm database. The *Synechococcus* 5′ UTR dataset was compiled from NCBI annotation. The 5′ UTR just prior to the start codon was considered in pieces: 18 bases prior, 40 bases prior, and 100 bases prior. It is notable that *E. coli* (top row and shown in gray in all other plots) contains fewer cytosines in its upstream region than the organisms which are susceptible to bacteriophage having C-rich 5′ UTRs.
(TIF)

Figure S5 Poly-A, poly-G, and poly-U RBS efficiency. (A) Single-clone ribosome display results with constructs containing poly-A, poly-G, or poly-U 18-base regions prior to the start codon are shown relative to the WT construct. Poly-G has even lower efficiency than poly-C (Figure 4), but poly-A and even poly-U are relatively efficient in a minimal, *E. coli*-based *in vitro* translation system. Error bars indicate the half range of duplicate wells. (B) The average median *in vivo* expression of emGFP (similar to that shown in Figure 5) from constructs containing a WT, poly-A, poly-G, or poly-U RBS is shown. The three homopolymer RBSs are more efficient than poly-C (Figure 5), but they are also much less efficient than WT *in vivo*. Error bars represent standard deviation of at least three independent experiments. MBP = maltose-binding protein.
(TIF)

Table S1 mRNA-rRNA complementarity. The first column on each worksheet provides the index of the first 16S rRNA base in the “motif” column. The incidence of complementarity in the data and *p*-values (P.rand, based on random null distribution; P.perm, based on permuted sequences as the null distribution) are also presented.
(XLS)

Table S2 Motif search results. The raw incidence and *q*-values (Q.rand, based on random sequences as the null distribution; Q.perm, based on permuted sequences as the null distribution) are presented.
(XLS)

Table S3 Co-occurrence of significant motifs. The number of sequences that contain both motif 1 and motif 2 is reported as “coincidence.” The co-occurrence metric is: coincidence/(motif 2 incidence).
(XLS)

Table S4 Oligonucleotide sequences.
(XLS)

Text S1 Supplemental Experimental Procedures.
(PDF)
