# Supplementary figures and images for: Broad-Specificity mRNA–rRNA Complementarity in Efficient Protein Translation
Source: PLoS Genet. 2012 Mar 22;8(3):e1002598. doi: 10.1371/journal.pgen.1002598 (PMC3310771; doi:10.1371/journal.pgen.1002598)

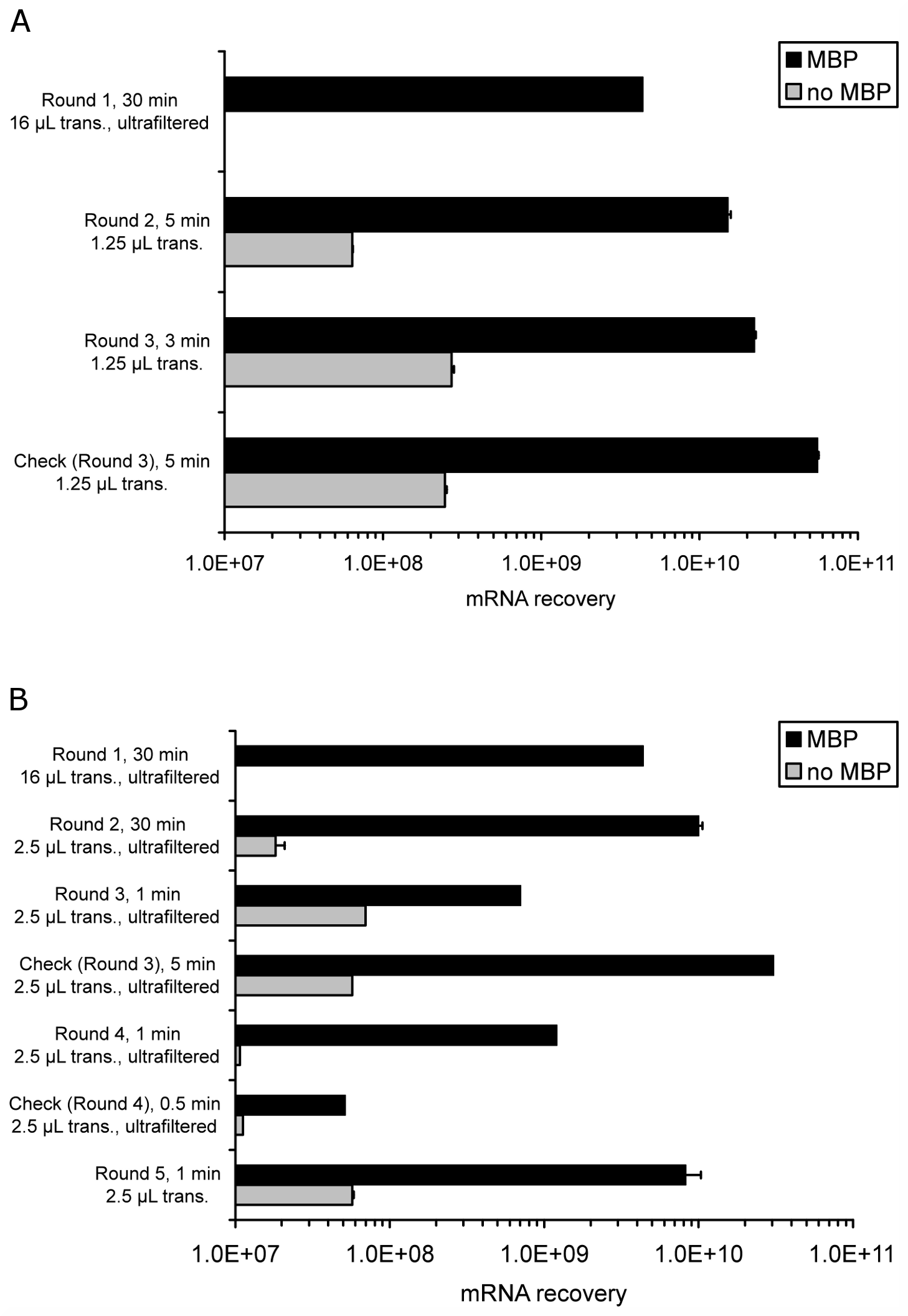

Supplement: Figure S1 — mRNA recovery. mRNA recovery was quantified by qRT-PCR after each round in (A) the basic 30-5-3 selection and (B) the alternate 30-30-1-1-1 selection. The translation time, translation volume, and ultrafiltration status are provided for each round. Where indicated, a “check” round was performed in parallel to the actual round to verify enrichment or to test a more stringent selection. In (A), the Round 3 check verified that enrichment had occurred between rounds 2 and 3. In (B), the Round 3 check verified enrichment, while the Round 4 check verified that an appropriate level of stringency had been applied. Error bars, when shown, indicate the half range of duplicate wells. The negative control (no MBP) was not performed in the first round. MBP = maltose-binding protein. (TIF) [file pgen.1002598.s001.tif]

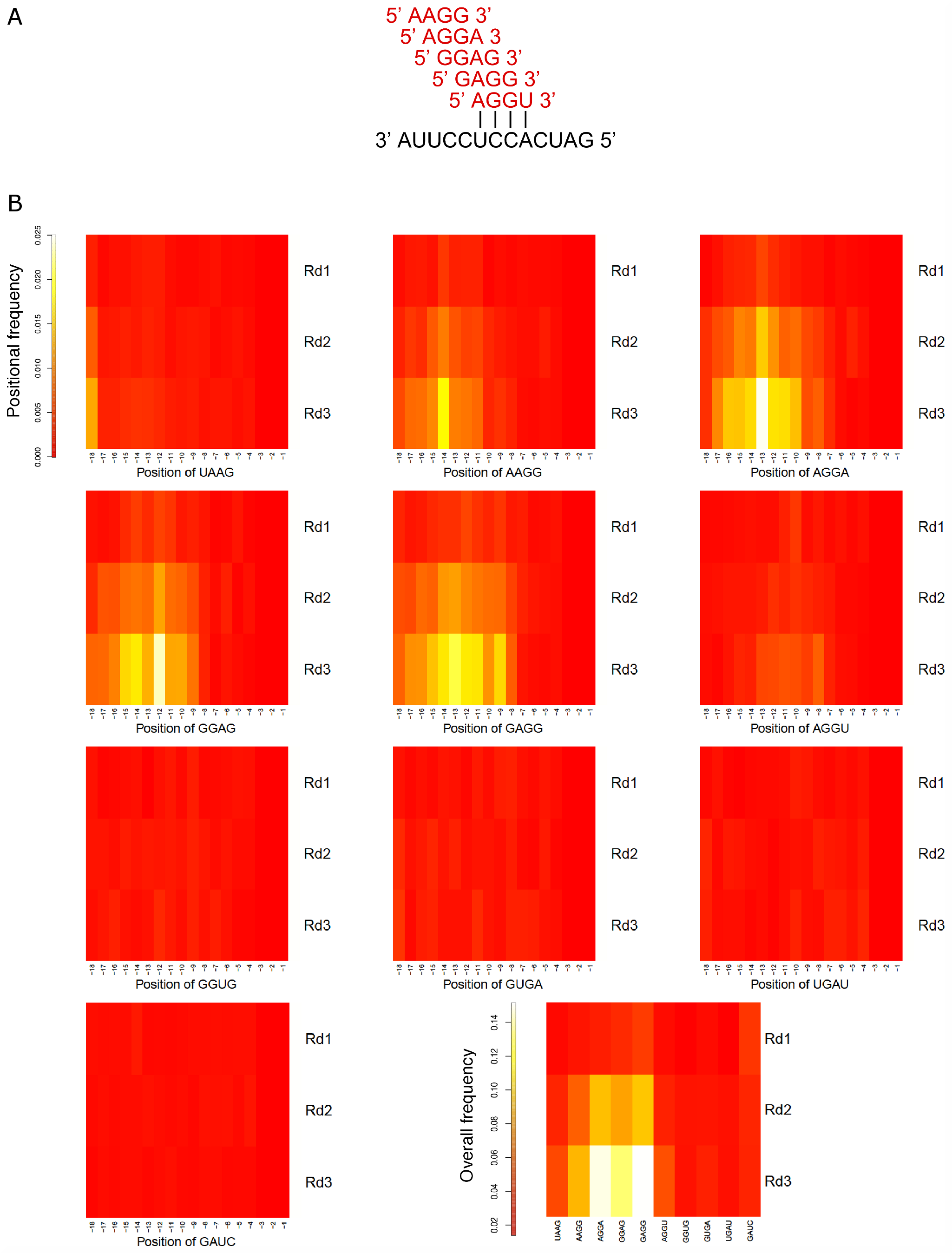

Supplement: Figure S2 — SD sequences in the 30-5-3 selection. (A) The alignment of study-defined SD motifs (red) with the 3′ tail of the 16S rRNA (black) is shown. (B) Position-dependent and overall enrichment of SD sequences over three rounds (Rd1, Rd2, Rd3) is shown. For comparison, we present all ten four-base subsets of the reverse complement (5′-UAAGGAGGUGAUC-3′) to the 13 unpaired bases at the 3′ end of the 16S rRNA (5′-GAUCACCUCCUUA-3′) in our selected sequences: UAAG, AAGG, AGGA, GGAG, GAGG, AGGU, GGUG, GUGA, UGAU, and GAUC. All SD motifs exhibited position-dependent enrichment according to their alignment with the 16S rRNA. (TIF) [file pgen.1002598.s002.tif]

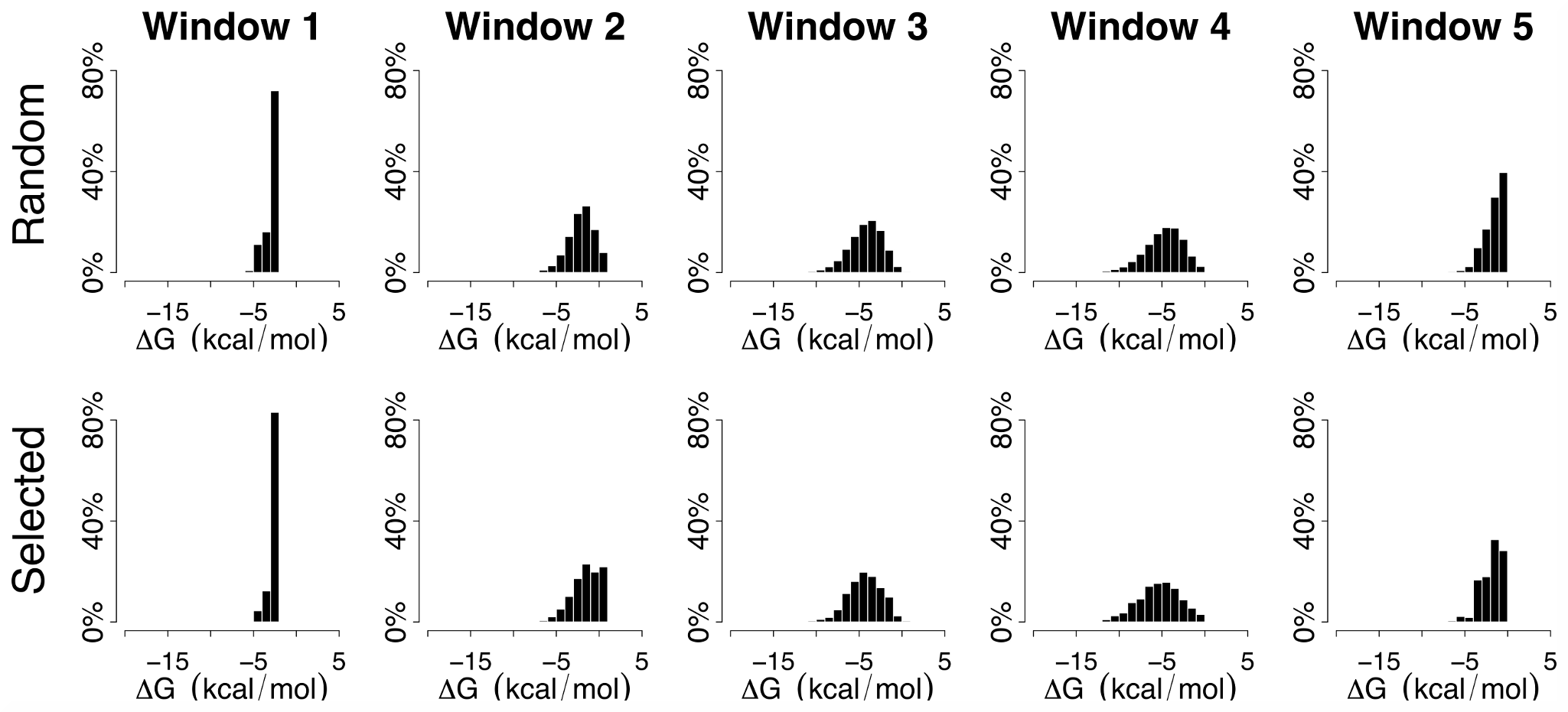

Supplement: Figure S3 — Histograms of ΔG values. Histograms of ΔG values in five 30-base sliding windows (offset by 10 bases) in a 70-base region centered on the 18-base randomized region in theoretical naïve (top) and selected (bottom) library from the basic selection are shown. The similarity of the distributions suggests no strong pressure for less or more secondary structure than a random library. (TIF) [file pgen.1002598.s003.tif]

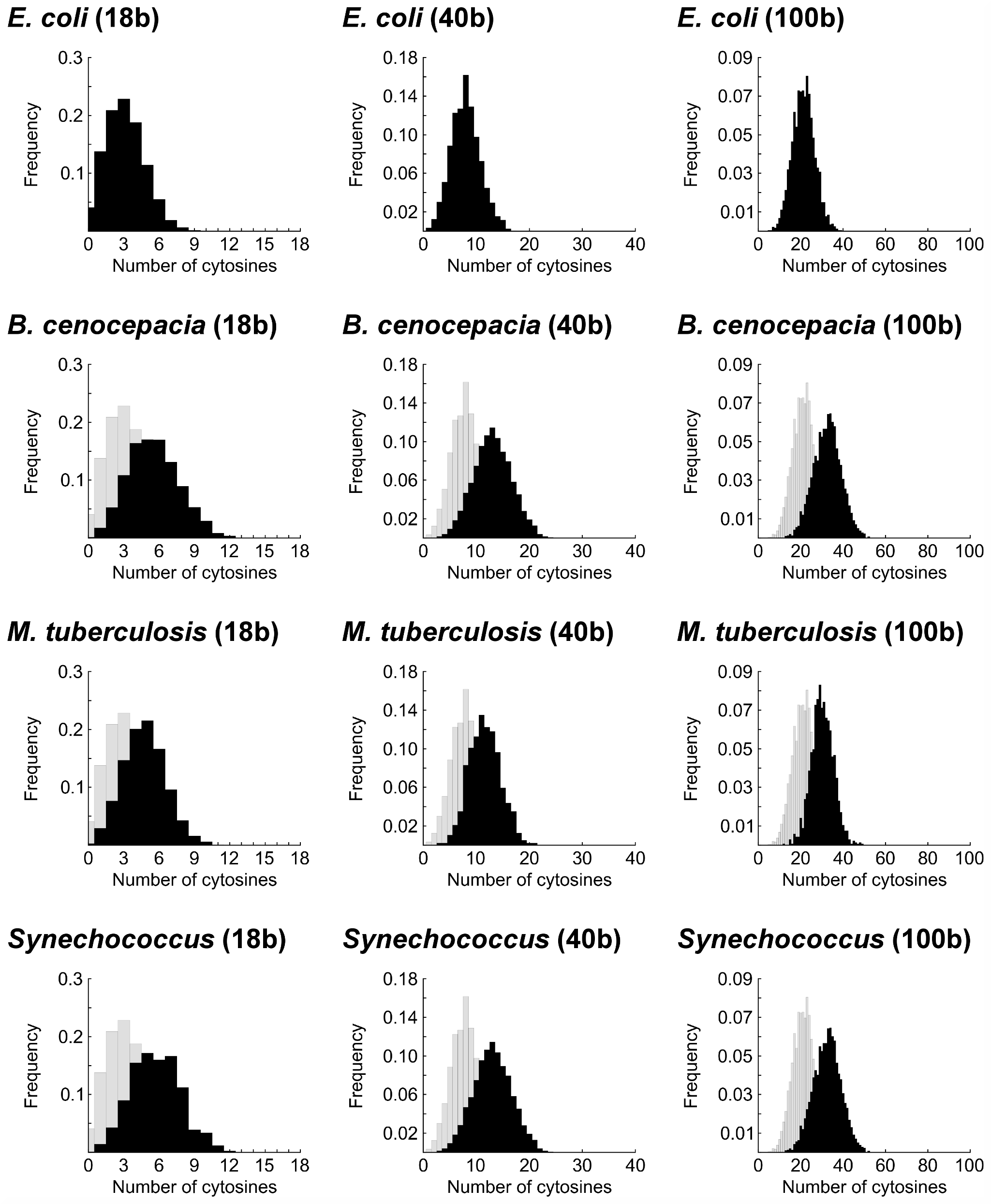

Supplement: Figure S4 — Histograms of natural cytosine content. Histograms of cytosine content in natural 5′ UTRs of E. coli K12 W3110 (NCBI TaxID: 316407) and three representative organisms that are infected by bacteriophage having very high cytosine content in at least one 5′ UTR (Burkholderia cenocepacia, TaxID: 331272, infected by Burkholderia phage KS14; Mycobacterium tuberculosis H37Rv, TaxID: 83332, infected by Mycobacterium phage Nigel; Synechococcus sp. WH 8109, TaxID: 166314, infected by Synechococcus phage Syn5) are shown. 5′ UTR datasets for all organisms except Synechococcus were obtained from the Transterm database. The Synechococcus 5′ UTR dataset was compiled from NCBI annotation. The 5′ UTR just prior to the start codon was considered in pieces: 18 bases prior, 40 bases prior, and 100 bases prior. It is notable that E. coli (top row and shown in gray in all other plots) contains fewer cytosines in its upstream region than the organisms which are susceptible to bacteriophage having C-rich 5′ UTRs. (TIF) [file pgen.1002598.s004.tif]

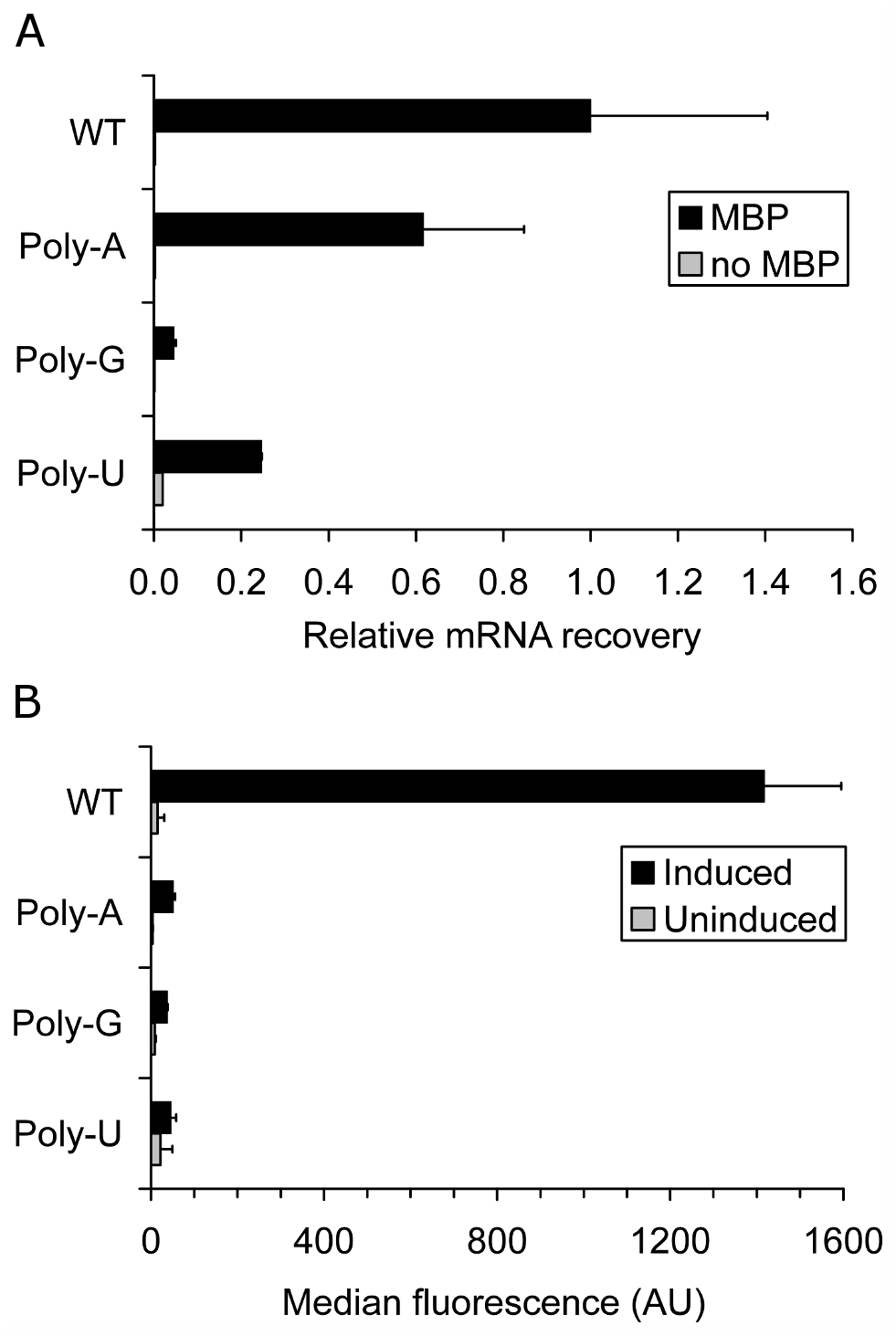

Supplement: Figure S5 — Poly-A, poly-G, and poly-U RBS efficiency. (A) Single-clone ribosome display results with constructs containing poly-A, poly-G, or poly-U 18-base regions prior to the start codon are shown relative to the WT construct. Poly-G has even lower efficiency than poly-C (Figure 4), but poly-A and even poly-U are relatively efficient in a minimal, E. coli-based in vitro translation system. Error bars indicate the half range of duplicate wells. (B) The average median in vivo expression of emGFP (similar to that shown in Figure 5) from constructs containing a WT, poly-A, poly-G, or poly-U RBS is shown. The three homopolymer RBSs are more efficient than poly-C (Figure 5), but they are also much less efficient than WT in vivo. Error bars represent standard deviation of at least three independent experiments. MBP = maltose-binding protein. (TIF) [file pgen.1002598.s005.tif]
