# Supplementary material for: Broad-Specificity mRNA–rRNA Complementarity in Efficient Protein Translation
Source: PLoS Genet. 2012 Mar 22;8(3):e1002598. doi: 10.1371/journal.pgen.1002598 (PMC3310771; doi:10.1371/journal.pgen.1002598)
Supplement: Text S1 — Supplemental Experimental Procedures. (PDF) [file pgen.1002598.s010.pdf]

Text S1

Broad-Specificity mRNA-rRNA Complementarity in Efficient Protein Translation

Pamela A. Barendt[1], Najaf A. Shah[2], Gregory A. Barendt[3], and Casim A. Sarkar[1,2,4]

[1]Department of Bioengineering, [2]Genomics and Computational Biology Graduate Group, [3]Perelman School of Medicine Information Services, [4]Department of Chemical & Biomolecular Engineering
University of Pennsylvania, Philadelphia, Pennsylvania 19104, USA

Supplemental Experimental Procedures

Detailed methods for ribosome display, single-clone ribosome display, *in vivo* experiments, and data analysis are given in the main text. Materials and additional procedures are provided below.

<u>Materials</u>

All oligonucleotides (Table S4) were purchased from Integrated DNA Technologies (Coralville, IA). All DNA purification was performed using agarose gel electrophoresis with SYBR Safe (Invitrogen, Carlsbad, CA) and QIAquick gel extraction kit (Qiagen, Valencia, CA) or with QIAquick PCR purification kit (Qiagen). All restriction enzymes, T4 DNA ligase (used for all ligations), and Phusion DNA polymerase (used for all PCRs) were purchased from New England Biolabs (Ipswich, MA).

<u>Library construction</u>

The library was constructed to minimize bias. First, a primer-extension product was made using two Ultramer oligonucleotides: T7_ext_fwd (5’-ATA CGA AAT TAA TAC GAC TCA

CTA TAG GGA CAC CAC AAC GGT TTC CCT AAT TGT GAG CGG ATA ACA ATA GAA ATA ATT TTG TTT AAC TT-3’) and BsaI_lib_rev (5’-ACT GAT TAG GTC TCG CCA TNN NNN NNN NNN NNN NNN NAA GTT AAA CAA AAT TAT TTC TAT T-3’). Underlined bases in T7_ext_fwd indicate minor changes to the published version of the ribosome display construct derived from the ribosome display vector pRDV [1]. The first change eliminates a BsaI site and the second is a mutation present in certain versions of the vector that more closely resembles the source sequence from phage. These were annealed and extended using the following program: 98°C for 1 min; 35 cycles of 47°C for 20 sec and 72°C for 4 sec; 72°C for 5 min; 4°C thereafter. The product was purified by agarose gel electrophoresis. Oligonucleotides BsaI_FLAG_fwd (5’-ACT GAT TAG GTC TCG ATG GCG GAC TAC AAA GAT G-3’) and tolAk (5’-CCG CAC ACC AGT AAG GTG TGC GGT TTC AGT TGC CGC TTT CTT TCT-3’) [1] were used to amplify FLAG-off7-tolA from pRDVstops-off7, a modified version of pRDV-off7 [1] which includes numerous out-of-frame stop codons in the *E. coli* tolA-derived spacer region between the gene of interest and the tolAk annealing site. Briefly, pRDVstops was constructed by removing the tolA region of pRDV-off7 by HindIII/EagI digestion and inserting a modified tolA built by assembly PCR. Primers HindIII_tolA_stops_fwd (5’- TAC TGC AAC AAG CTT GGA TCT GGT GGC CAG AA-3) and tolA_stops1_rev (Table S4) were used to make the first piece; primers tolA_stops2_fwd (Table S4) and EagI_rev (5’- CTG CCT CTG CGG CCG C-3’) were used to amplify the tolA region from pRDV to make the second piece. These products were purified and assembled by PCR. BsaI_FLAG_fwd/tolAk-amplified FLAG-off7-tolA was purified by agarose gel electrophoresis. The T7_ext_fwd/BsaI_lib_rev primer-extension product (126 bp) and FLAG-off7-tolA (807 bp) were separately digested with BsaI to create compatible sticky ends (5’-CCAT-3’ and 5’-ATGG-3,’ respectively). The digests were

purified and ligated at 16°C for 90 min. The full-length product (899 bp) was gel-purified according to Qiagen, except that RNase-free water was used for elution instead of EB buffer. This product was used directly for transcription, as previously described [2].

Single-clone ribosome display constructs

DNA constructs of some clones were built to verify RBS activity. Generally, a forward primer encoding a constant 5’ piece, the clone of interest, and an annealing region (i.e., 5’-TGA GCG GAT AAC AAT AGA AAT AAT TTT GTT TAA CTT XXX XXX XXX XXX XXX XXX ATG GCG GAC TAC AAA GAT GAC-3’ where the Xs correspond to a specific clone) was used in conjunction with tolAk to create a product containing the RBS of interest, off7, and tolA. The specific sequences of these primers are listed in Table S4 (30-30-1-1_high_C_clone_1 through 30-5-3_high_C_clone_11). This downstream piece was assembled by PCR with an upstream piece containing the 5’ region of pRDV, which was made separately by PCR using T7_no_BsaI (5’-ATA CGA AAT TAA TAC GAC TCA CTA TAG GGA CAC CAC AAC GG-3’) and 5’_UTR_rev (5’-AAG TTA AAC AAA ATT ATT TCT ATT GTT ATC CGC TCA-3’). An alternate strategy was used for the homopolymers, which employed T7_no_BsaI and a reverse primer (e.g., poly-A_rev: 5’-ACT GAT TAG GTC TCG CCA TTT TTT TTT TTT TTT TTT TAA GTT AAA CAA AAT TAT TTC TAT-3’) to amplify the 5’ UTR of pRDV. The BsaI digestion product was ligated to FLAG-off7-tolA as in the original library construction. Problems with synthesizing poly-G oligos required us to use poly-C_fwd (5’- ACT GAT TAG GTC TCG TTC CCC CCC CCC CCC CCC CCA TGG CGG ACT ACA AAG ATG-3’) and tolAk to amplify FLAG-off7-tolA, while T7_no_BsaI and BsaI_5'_UTR_rev (5’- ACT GAT

TAG GTC TCG GGA AGT TAA ACA AAA TTA TTT CTA TT-3') were used to amplify the 5' UTR. Both pieces were digested with BsaI and then ligated.

The assembled product was re-amplified with BsaI_GATC_T7_fwd (5'-TGT AAT AAG GTC TCG GAT CCA TAC GAA ATT AAT ACG ACT CA-3') and BsaI_AGCT_tolA_rev (5'-TGT AAT AAG GTC TCA AGC TTC AGT TGC CGC TTT C-3'), digested with BsaI to make BamHI- and HindIII-compatible sticky ends, and cloned into pUC19 between BamHI and HindIII.

In vivo expression constructs

pET-3a-RBS-FLAG-off7-emerald GFP (emGFP) constructs were made by first assembling the insert by PCR. BglII_5'_UTR_fwd (5'- ACT GAT TAA GAT CTC GAT CCC GCG AAA TTA ATA CGA CTC ACT ATA GGG ACA CCA CAA CGG-3') and 5'_UTR_rev were used to amplify the 5' UTR of pRDV. A clone-specific forward primer (i.e., 5'-TGA GCG GAT AAC AAT AGA AAT AAT TTT GTT TAA CTT XXX XXX XXX XXX XXX XXX ATG GCG GAC TAC AAA GAT GAC-3' where the Xs correspond to a specific clone; E._coli_thiI_5'_UTR through Phage_high_C_clone_3 in Table S4) and early_tolA_rev (5'- CGC CTT AGC TGC CGC CTC CTC AGC-3') were used to amplify RBS-FLAG-off7 from pRDVstops-off7. Finally, early_tolA_emGFP_fwd (5'-GCT GAG GAG GCG GCA GCT AAG GCG GTG AGC AAG GGC GAG GAG-3') and BlpI_emGFP_rev (5'- TAG TTA TTG CTC AGC TTA CTT GTA CAG CTC GTC CAT-3') were used to amplify emGFP from pRSET-emGFP (Invitrogen). These three products were subjected to 10 cycles of overlap PCR and then amplified with BglII_5'_UTR_short_fwd (5'-ACT GAT TAA GAT CTC GAT CCC GCG AAA TT-3') and BlpI_emGFP_short_rev (5'- TAG TTA TTG CTC AGC TTA CTT GTA CAG CT-

3’) for an additional 30 cycles. (For WT, poly-C, poly-A, poly-G, and poly-U, BglII_5’_UTR_fwd and early_tolA_rev were used to amplify RBS-FLAG-off7 from existing plasmids, so only two pieces were assembled in these cases.) The assembled products were cloned into pET-3a (EMD Serono, Rockland, MA) between BglII and BlpI.

References

1. Binz HK, Amstutz P, Kohl A, Stumpp MT, Briand C, et al. (2004) High-affinity binders selected from designed ankyrin repeat protein libraries. Nat Biotechnol 22: 575–582. doi:10.1038/nbt962.

2. Dreier B, Plückthun A (2011) Ribosome display: a technology for selecting and evolving proteins from large libraries. Methods Mol Biol 687: 283–306. doi:10.1007/978-1-60761-944-4_21.
